# Fully 3D Active Surface with Machine Learning for PET Image Segmentation

**DOI:** 10.3390/jimaging6110113

**Published:** 2020-10-23

**Authors:** Albert Comelli

**Affiliations:** Ri.MED Foundation, 90133 Palermo, Italy; acomelli@fondazionerimed.com; Tel.: +39-3333967105

**Keywords:** 3D segmentation, machine learning, active surface, discriminant analysis, PET imaging

## Abstract

In order to tackle three-dimensional tumor volume reconstruction from Positron Emission Tomography (PET) images, most of the existing algorithms rely on the segmentation of independent PET slices. To exploit cross-slice information, typically overlooked in these 2D implementations, I present an algorithm capable of achieving the volume reconstruction directly in 3D, by leveraging an active surface algorithm. The evolution of such surface performs the segmentation of the whole stack of slices simultaneously and can handle changes in topology. Furthermore, no artificial stop condition is required, as the active surface will naturally converge to a stable topology. In addition, I include a machine learning component to enhance the accuracy of the segmentation process. The latter consists of a forcing term based on classification results from a discriminant analysis algorithm, which is included directly in the mathematical formulation of the energy function driving surface evolution. It is worth noting that the training of such a component requires minimal data compared to more involved deep learning methods. Only eight patients (i.e., two lung, four head and neck, and two brain cancers) were used for training and testing the machine learning component, while fifty patients (i.e., 10 lung, 25 head and neck, and 15 brain cancers) were used to test the full 3D reconstruction algorithm. Performance evaluation is based on the same dataset of patients discussed in my previous work, where the segmentation was performed using the 2D active contour. The results confirm that the active surface algorithm is superior to the active contour algorithm, outperforming the earlier approach on all the investigated anatomical districts with a dice similarity coefficient of 90.47 ± 2.36% for lung cancer, 88.30 ± 2.89% for head and neck cancer, and 90.29 ± 2.52% for brain cancer. Based on the reported results, it can be claimed that the migration into a 3D system yielded a practical benefit justifying the effort to rewrite an existing 2D system for PET imaging segmentation.

## 1. Introduction

In oncological studies, the main motivation to image segmentation is to recognize the portions of ill tissues so that treatment can be better directed (e.g., radio-therapy, [1,2,3]) and chances of survival improved [4,5,6,7]. Furthermore, segmentation is a pre-requisite to advanced studies such as radiomics feature extraction [8,9,10]. The present study discusses the segmentation of Positron Emission Tomography images leveraging a fully 3D hybrid algorithm which combines traditional segmentation (e.g., active surface) with machine learning (ML). Unlike several algorithms based on the segmentation of independent Positron Emission Tomography (PET) slices, the present algorithm is capable of segmenting all the slices in a PET data set simultaneously with the immediate advantage of exploiting valuable cross-slice information which would be ignored otherwise (e.g., [11,12,13]). In addition, in order to improve the accuracy of the final result, I included a machine learning component. A term based on information from a classifier (i.e., discriminant analysis) is included in the formulation of the energy function driving the surface evolution. The main advantage brought by artificial intelligence is that these approaches are designed to “learn” a non-obvious input–output relation directly from data. In the clinical context, artificial intelligence is used to obtain the morphology of organs or for the delineation of pathological tissue. Examples include the identification of cancer within the head and the neck using deep learning [14,15], classification using a kernel support vector machine [16], thyroid volume computation in 3D ultrasound imaging using random forest and convolutional neural network (CNN) [17], etc. It could be observed that deep learning techniques are more efficient than classical statistical approaches and, consequently, they are applied in many biomedical image segmentation tasks [18]. Nevertheless, while such methods are commonly used in magnetic resonance or computerized tomography imaging, their application on PET is still rather scarce and not much is found in the literature [19]. The reason is that in order to be successful, deep learning approaches require datasets containing hundreds of labelled examples, which is rarely the case in nuclear medicine. Vice versa, deep learning is largely used to classify patients in terms of outcome based on quantitative features extracted from previously delineated tumors for radiomics analyses of PET images [20]. Furthermore, to date, artificial intelligence algorithms are often employed as a stand-alone tool. Conversely, such a class of algorithms can be leveraged to improve the performance of more traditional approaches as well. For example, in [11], the k-means clustering technique was used to support a random walk segmentation algorithm, while, in [21], a convolutional neural network was used to obtain an initial delineation, successively refined by threshold segmentation and morphological methods. Moreover, an enhanced active contour (AC) algorithm has been proposed to delineate tumor in PET datasets in a semi-automatic way [22]. In detail, Comelli et al. [22] developed a 2D algorithm based on an AC enhanced by the inclusion of tissue classification information [23,24,25]. In these studies, authors reconstructed the 3D tumor shape by separately segmenting 2D PET slices because the main goal was to obtain a delineation method capable of being efficient, repeatable and real time. The core of such volume reconstruction was an active contour segmentation of PET images (i.e., 2D), performed marching through the PET slices and where the converged segmentation of one slice was used to initialize segmentation on the next. Nevertheless, the distance between consecutive PET slices is typically much greater than their voxel size. This feature introduces a sort of preferential direction in the data which may influence the reconstructed volume. In addition, the 2D contour evolution on one PET slice does not retain memory of what actually happened in the previous slice, nor does it takes into account what is the information in the slice that will come next. 

Based on these considerations, the present study proposes a fully 3D active surface (AS) driven by a 3D machine learning component (i.e., 3D tissue classification). Starting from a region placed around the target, the AS deforms and moves to fit the target minimizing an “energy”, defined as a multi-parameter function. In addition, the ML component, trained trough examples generated by expert human operators, learns to classify the PET data (i.e., the voxels in the slices) and introduces insight into the AS on which tissue portions are to be considered tumor or healthy. Therefore, to some extent, the algorithm learns to “mimic” how a human would perform this complex task and to avoid false positives. Although several classifiers were considered in previous publications (k-nearest neighbors, Naive Bayes, and Discriminant Analysis [23,24,25]), it is worth noting that the discriminant analysis provided consistently better performances [24]. As such, since a quite large comparative work was performed in [23,24,25], and since a full investigation on which classification approach would perform best within the new 3D framework is beyond the scope of the present study, the discriminant analysis is the algorithm of choice for this publication as well. 

Summarizing, the key aspect of the proposed study is the evolution of a 3D surface embedded in the data volume, henceforth referred to as active surface, the evolution of which is driven by the minimization of a cost function specifically built to take into account information contained in the data and from 3D tissue classification. To evaluate the performance of the algorithm, fifty-eight patient studies are considered. In detail, eight studies are used for training and testing of the artificial intelligence component and fifty to test the full 3D reconstruction algorithm.

## 2. Materials and Methods 

### 2.1. Overview of the 3D as Proposed Method and Differences with the Previous 2D AC Method

**Figure 1:**
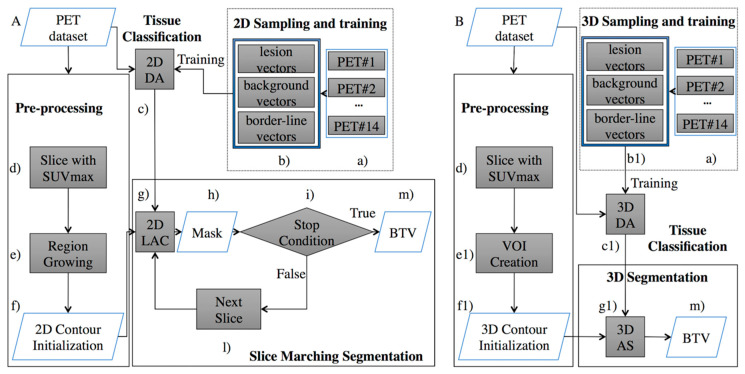
Comparison of the Positron Emission Tomography (PET) system proposed in the previous work (**A**) [24], and the implementation set out here (**B**). The proposed implementation substitutes the ‘Slice Marching Segmentation’ block (**A**) with the fully “3D Segmentation” block (**B**). Therefore, cross-slice information previously ignored is now being completely exploited. Moreover, the artificial stopping condition (step i) is no longer necessary. Additionally, (steps b, c, f, and g) were modified in order to provide a 3D sampling and training (step b1), a 3D tissue classification (step c1), 3D contour initialization (step f1), and 3D AS (step g1).

This study proposes a re-engineering of the algorithm from my previous work and focuses on the development of a 3D AS for the volume delineation augmented with the inclusion of a discriminant analysis (DA) component for 3D tissue classification. Figure 1 compares the 2D DA system proposed in [24] and the new 3D DA implementation discussed here to highlight differences and improvements.

Figure 1A describes the system from my previous work [24]. Here, the “Pre-processing” block consisted of a pre-segmentation where the user draws, on a single PET slice, a contour roughly encircling the cancer area. This input is required to minimize the risk of incurring in false positives. Such an input was used to generate a user-independent mask which could be located on a different slice from the one chosen by the operator. By construction, this mask belonged to the same anatomical anomaly and included the radio-tracer maximum absorption area. After this step, the initial mask was fed to the next block of the workflow (“Slice Marching Segmentation” step), where the segmentation was obtained using a local region-based AC in which the contour evolved while minimizing its energy function. Information from the “2D Sampling and training” block consisted of labels associated to the PET image’s pixels which identified each pixel as “lesion”, “background”, or “border-line” tissue. The different tissues in the PET slices were automatically identified using a 2D floating window (3 × 3 voxels). Finally, this algorithm used a slice-by-slice marching strategy to obtain an accurate segmentation, which began at the slice containing the user-independent mask and propagated upward and downward. Every time a slice segmentation was completed, the contour was propagated to the nearest adjacent slice until the stop condition was finally met. The final 3D shape consisted of the union of all contours obtained on each slice.

The algorithm presented here (Figure 1B) inherits the same pre-segmentation workflow of [24], although a 3D shape is required as the output of the pre-segmentation step. Specifically, to initialize the AS (step “g1” in Figure 1B), the pre-segmentation step (step “f1” in Figure 1B) must output a 3D shape transforming the user independent mask (step “f” in Figure 1A) in an ellipsoid (the 3D shape needed to start the 3D AS method). It is worth noting that the only requirement for the ellipsoid is that it envelops a portion of the tumor, including the voxel with the maximum intensity value (requirement already satisfied in [24] and naturally inherited from the ellipsoid). The energy function being minimized depends on the shape of the curves generated by the intersection of the AS with the planes containing the PET slices. Consequently, the ellipsoid around the tumor changes shape and moves towards the tumor borders with the aim of minimizing the energy function. Information from the 3D classifier is now included in the energy function in terms of labelled voxels combining, during the classification process, information involving multiple slices. As in the previous 2D classifier [24], the proposed 3D classifier labels the tissue as “lesion”, “background” or “border-line” and drives the 3D AS algorithm (“3D Sampling and training” block), although the classification occurs now in terms of voxels and not pixels. This task comprises the training and validation steps of the DA classifier. Different tissues (i.e., lesion, background, or border-line tissue) are automatically identified in the PET data volume using a 3D floating window (i.e., 3 × 3 × 3 voxels). The highlighted values are then arranged for every window into 27-element vectors describing the volume sample being investigated. The “training and testing” step needs to be performed only once. When completed, the classifier is capable of labelling new tissue and, subsequently, this information will be used to guide the segmentation process. A thorough explanation of this aspect will be provided in Section 2.1.1. The main advantage of leveraging a AS is that it is free to intersect the whole volume of PET images (i.e., all slices) and therefore segments them all at once, using cross-slice information, both in terms of data volume and tissue labels, that could not be leveraged before (i.e., in the system of Figure 1A). Details of this part will be given in Section 2.1.2. Finally, an operator-independent biological tumor volume (BTV) is obtained (step “m” in Figure 1B). 

The following subsections will provide detailed discussion of the various components of system 1-A.

#### 2.1.1. The 3D Discriminant Analysis

The DA was used to classify PET tissues into three clusters: background, lesion, and border-line tissues. The training needs to be done only once, after which DA is able to classify newly encountered tissues and can be re-used at any time. 

Three clinicians with different expertise segmented 58 patients [24] (see Section 2.2 for more details). The STAPLE tool [26] was used to combine the three manual segmentations and to define a consolidated reference to be used as gold standard. Only eight patients (considering multiple PET slices for each study) were used for the training and testing of the 3D tissue classification algorithm. These comprised 2 with brain metastases, 4 with head and neck cancers, and 2 with lung cancers, respectively. The sampling process produced 13,762,560 vectors divided into 28,152 lesion vectors, 16,107 border-line vectors, and 13,718,301 background vectors. Specifically, in order to extract such samples, each PET slice was processed using a 3D floating window (i.e., 3 × 3× 3 voxels). The values in every window were arranged into 27-element vectors. Such a sample size was empirically determined to provide the best results on the current data set.

Of the generated samples, 80% were used for the training step, while the remaining 20% were used for the testing step (as stated by the Pareto principle, also known as the 80/20 rule). The K-Fold cross-validation was used to obtain a robust classification and to eliminate over-fitting. Accordingly, the dataset was split into K equal folds, and the holdout method repeated K times. Every time one of the K folds was used as a validation set, and the remaining K-1 subsets were assembled to form a training set. The variance of the resulting estimate decreased as K increased. Samples with no more than 17 lesion voxels or totally outside the gold standard were labelled as “border-line” and “background” tissue, respectively. In all other instances, the label “lesion” was assigned. Once the training process was completed, a validation stage to test the efficiency of the classification followed.

#### 2.1.2. The Active Surface Algorithm

Starting from the model proposed by Lankton et al. [27], which benefits purely local edge-based active contours and more global region-based active contours, I propose a more efficient segmentation technique that segments all slices simultaneously by developing a single surface inside the corresponding 3D PET volume [28], and a new mathematical formulation of the active surface energy including the 3D classifier information. 

Summarizing, the obtained algorithm possesses the following novelty and improvements:

- A new active surface volumetric energy formulation.
- A full 3D development so leveraging cross-slice information.
- Inclusion of 3D tissue information.

These aspects are further detailed below. 

First, a new formulation of the energy driving the AS by the inclusion of information from the 3D classifier is introduced. 

In view of the fact that the tumor and the background regions are not easily split into two distinct regions, the energy was modified to include a new energy term based on tissue classes generated by the 3D classifier:

(1) $$\chi_{lesion}\left(x\right)=\{\begin{array}{l} 1\;\;\;\;\;\;when\;\;\;3D\;DA\left(x\right)=\;lesion;\\ 0\;\;\;\;\;\;\;\;\;\;\;\;\;\;\;\;\;\;\;\;\;\;\;\;\;\;\;otherwise;\;\;\;\;\;\;\;\;\;\;\; \end{array}$$

(2) $$\chi_{border-line\;}\left(x\right)=\{\begin{array}{l} 1\;\;\;\;\;\;\;when\;\;\;3D\;DA\left(x\right)=\;borderline\;;\\ 0\;\;\;\;\;\;\;\;\;\;\;\;\;\;\;\;\;\;\;\;\;\;\;\;\;\;\;\;\;\;\;\;\;\;\;\;\;\;\;otherwise;\;\;\;\;\;\;\;\;\;\;\; \end{array}$$

(3) $$\chi_{\mathrm{background}}\left(x\right)=\{\begin{array}{l} 1\;\;\;\;\;\;when\;\;\;3D\;DA\left(x\right)=\;background;\\ 0\;\;\;\;\;\;\;\;\;\;\;\;\;\;\;\;\;\;\;\;\;\;\;\;\;\;\;\;\;\;\;\;\;\;\;\;\;\;\;otherwise;\;\;\;\;\;\;\;\;\;\;\; \end{array}$$

where the characteristic functions $\chi_{\mathrm{lesion}}\left(x\right)$, $\chi_{\mathrm{border}-\mathrm{line}}\left(x\right)$ and $\chi_{\mathrm{background}}\left(x\right)$ represent the 3D tissue’s classification for lesion, border-line, and background, respectively.

In Functional Energy Equation (4), the first term is basically a prior term penalizing the overlap between regions which are classified in a conflicting way by the 3D AS and the 3D classifier (in regions classified as “border-line” no penalty is applied). 

To integrate the standardized uptake value (SUV) and to insert this new prior term in the mathematical model and formulate it in a fully 3D setup, the active surface energy to be minimized for PET image segmentation is defined as:

(4) $$E=\overset{\;}{\underset{S}{\int }}\;\lambda \left(\overset{\;}{\underset{R_{in}}{\int }}\chi_{l}\left(x,s\right){\bar{P}\mathrm{out}}_{l}\left(x\right)\;dx\;+\overset{\;}{\underset{R_{out}}{\int }}\chi_{l}\left(x,s\right){\bar{P}\mathrm{in}}_{l}\left(x\right)\;dx\right)+\;\left(1-\lambda \right)\left(\overset{\;}{\underset{R_{in}}{\int }}\chi_{l}\left(x,s\right){\left(\mathrm{SUV}\left(x\right)-u_{l}\left(s\right)\right)}^{2}dx+\overset{\;}{\underset{R_{out}}{\int }}\chi_{l}\left(x,s\right){\left(\mathrm{SUV}\left(x\right)-v_{l}\left(s\right)\right)}^{2}dx\right)dS$$

- $\lambda \in R^{+}$ was chosen equal to 0.01, because such value proved to yield the best result;
- ${\bar{P}\mathrm{in}}_{l}\left(x\right)$ and ${\bar{P}\mathrm{out}}_{l}\left(x\right)$ indicate the 3D local mean DA classification within the portions of the local neighborhood $\chi_{l}\left(x\right)$ inside and outside the surface, respectively (within Ω);
- *S* is the 3D AS and *dS* is the surface area measure;
- *s* is the 3D surface parameter;
- *x* is a point within the 3D volume and *dx* the volume measure;
- *R_in_* and *R_out_* are the corresponding 3D regions inside and outside the surface;
- $\chi_{l}(x,s$) is the indicator function around the surface point S(s) of a local neighborhood.

Such neighborhoods are spheres of radius l centered around each point of the surface S. It was equal to 3 as proposed in [19].

(5) $${\bar{P}\mathrm{in}}_{l}\left(x\right)=\;\frac{\int_{\Omega }^{\;}\chi_{l}\left(x\right)\chi_{lesion}\left(x\right)\;dx}{\int_{\Omega }^{\;}\chi_{l}\left(x\right)\;dx}\;,\;\;{\bar{P}\mathrm{out}}_{l}\left(x\right)=\;\frac{\int_{\Omega }^{\;}\chi_{l}\left(x\right)\chi_{\mathrm{background}}\left(x\right)\;dx}{\int_{\Omega }^{\;}\chi_{l}\left(x\right)\;dx}$$

In practice, the 3D surface obtained after pre-segmentation is used as initialization to the AS and evolved to minimize the energy E until it finally fits the tumor silhouette.

### 2.2. The Clinical Dataset 

In order to compare the performance of this new algorithm to its 2D predecessor, I considered the same dataset discussed in [24], which comprises 12 lung, 29 head and neck, and 17 brain cancers for a total of 58 PET studies. Eight PET datasets (2 with brain metastases, 4 with head and neck cancers, 2 with lung cancers) were used to train and test the 3D DA classifier while the remaining fifty PET datasets (i.e., 10 lung, 25 head and neck, and 15 brain cancers) were used to evaluate the performance of the proposed segmentation method. 

Segmentations were done off-line and the findings did not affect either the treatment plan or the care of the patient. No personal information on patients has been released. Concerning the use of dataset, written consent was released by patients. 

The PET investigation protocol was approved by the institutional Hospital Medical Ethics Review Board. Acquisitions were performed on a Discovery 690 PET/CT scanner with time of flight (General Electric Medical Systems, Milwaukee, WI, USA) and produced PET images with resolution of 256 × 256 voxels with grid spacing of 2.73 mm^3^, thickness of 3.27 mm^3^ and voxel size 2.73 × 2.73 × 3.27 mm^3^. 

### 2.3. Framework for Performance Evaluation

The following parameters are used here for performance evaluation:

(6) $$Sensitivity=\;\frac{True\;Positive}{True\;Positive+False\;Negative}$$

(7) $$Specificity\;=\;\frac{True\;Negative}{True\;Negative+False\;Positive}$$

(8) $$positive\;predictive\;value\;\left(PPV\right)\;=\;\frac{True\;Positive}{True\;Positive+False\;Positive}$$

(9) $$Accuracy\left(ACC\right)\;=\;\frac{True\;Positive+True\;Negative}{True\;Positive+False\;Positive+False\;Negative+True\;Negative}$$

(10) $$Dice\;similarity\;coefficient\;\left(DSC\right)=\;\frac{2\times True\;Positive}{2\times True\;Positive+False\;Positive+False\;Negative}$$

(11) Hausdorff distance (HD) =max{d(A,B),d(B,A)}

(12) $$Pearson\;correlation\;coefficient\;\left(PCC\right)=\;\frac{COV\left(X,Y\right)}{\sigma X\sigma Y}$$

The PCC can take a value between +1 and −1, where +1 and −1 show total correlation (no difference between two segmentations), while 0 means no correlation (totally difference between two segmentations).

The proposed fully 3D DA segmentation method was implemented in Matlab R2019a and run on MacBook Pro computer with a 2.5 GHz Intel Core i7 processor, 16 GB 1600 MHz DDR3 memory, and OS X Sierra.

## 3. Results

### Segmentation Results

**Figure 2:**
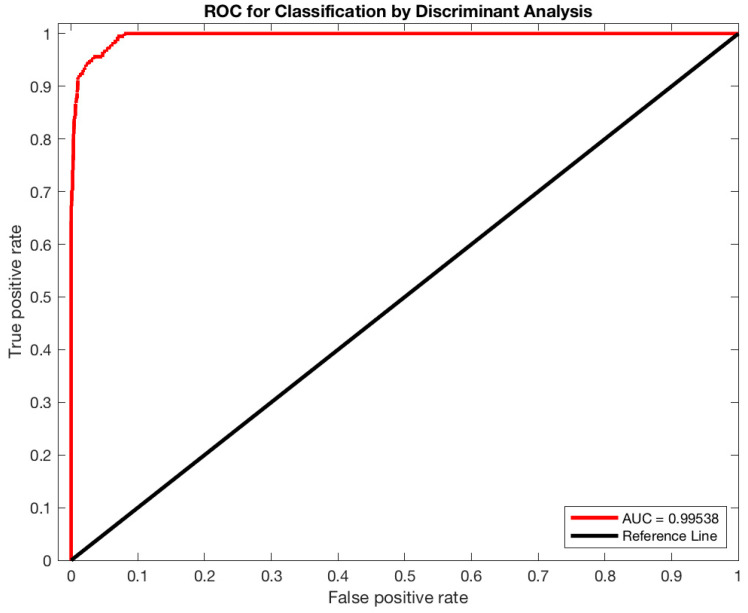
Classification performance of the 3D DA classifier was evaluated by calculating the area under the receiver operating characteristic (ROC) curve (AUC) using the ROC analysis.

To train and test the classifier, tumor, background and border-line tissues were automatically identified on PET images using the strategy described in Section 2.1.1. Starting from eight patient cases, the sampling process produced 13,762,560 vectors divided into 28,152 lesion vectors, 16,107 border-line vectors, and 13,718,301 background vectors. In total, 80% of the samples were used for training, while the remaining 20% were used for testing. Concerning the K-Fold cross-validation, the optimal k value was determined through the trial-and-error method (k ranged: 5–15, step size of 5), k = 5 corresponded to the highest classification accuracy. The capability of the 3D DA classifier in discerning different tissues achieved excellent result with a sensitivity of 99.09%, a specificity of 90.58%, a precision of 98.53%, and an accuracy of 96.46%. Figure 2 shows the receiver operating characteristic (ROC) analysis performed to assess the efficiency of 3D DA classification after the training phase. The area under the curve (AUC) is 0.99538. The maximum AUC is 1, which corresponds to the perfect classifier. After this training, the classifier is able to label new tissues and is ready to be integrated in the segmentation process (as described in Section 2.1.2).

To assess the performance of the segmentation method, ten patient studies with lung cancer (^18^F-fluorodeoxyglucose PET), 25 with head and neck cancer (^18^F-fluorodeoxyglucose PET), and 15 with brain cancer (^11^C-methionine PET) were considered. Table 1 summarizes the results of the three previous anatomical districts including the results from the previous study [24]. Since the 2D slice-by-slice approach worked better than several other state-of-the-art approaches [24], I felt judged comparison with additional approaches to be unnecessary.

As confirmed by the analysis of variance (ANOVA) (see Table 2), the proposed 3D AS DA algorithm generated better results than the previous 2D algorithm. This was further demonstrated by the statistical difference (ANOVA *p*-value < 0.047) in the DSC comparison. In addition, Pearson correlation coefficient (PCC) analysis has been performed to evaluate the similarity of the proposed segmentation with the previous method [24], across all the fifty cases. The fully 3D AS DA approach, with a mean DSC of 89.69 ± 1.21%, not only performed overall better than the old method (DSC = 87.24 ± 4.59%), but with PCC~0.53 provided significantly different results as well (*p*-value = 0.000044).

**Figure 3:**
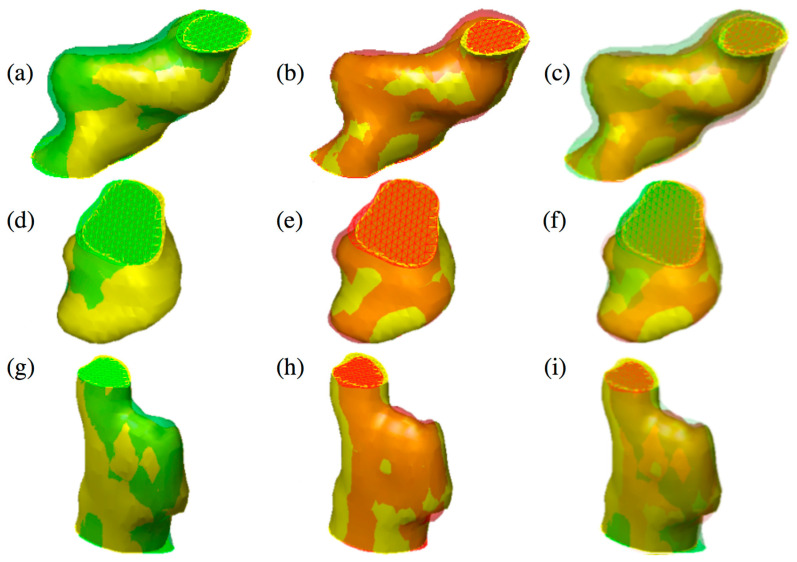
Three different tumors reported in the figure show the difference between 2D active contours (**a**,**d**,**g**) [24] and 3D active surface (**b**,**e**,**h**) segmentations guided by the 3D classifier. The reconstructed surfaces (green and red) and the gold standards (in yellow) are rendered partially transparent for better comparison. In the last column, the overlap of both methods and the gold standard is shown (**c**,**f**,**i**). Specifically, an over-segmentation of the 2D approach compared to 3D can be observed. (Color images can be found on the electronic version of this article).

Figure 3 shows the qualitative comparison between the proposed method and the algorithm proposed in [24].

## 4. Discussion

In this study, I discuss the inclusion of 3D tissue labelling with in the energy to be minimized during the 3D segmentation based on AS. The key improvement over the previous work [24] is a new segmentation algorithm obtained by evolving a 3D active surface, not only according to the PET data itself, but also leveraging information conveyed by a purposely-trained 3D DA classifier. Such information is included directly in the energy formulation. Previously [24], the 3D shape reconstruction challenge was addressed by using a reconstruction strategy based on a 2D approach, where the tumor segmentation was performed on each slice independently, and marching from one slice to the next until a stop condition is satisfied. While this represented an important step toward 3D delineation, it introduced a preferential direction (the direction of the marching) in the algorithm. In addition, the presence of a preferential direction is an embedded feature of PET data itself, being the distance between pet slices much greater than their planar resolution. While this particular aspect did not seem to influence the outcome of [24], it is nevertheless possible to conceive tumor topologies for which the marching 2D algorithm could actually ignore part of the tumor. To remove such a risk, I wanted to perform the shape reconstruction using an algorithm capable of handling any topology and where no preferential direction is present, either implicitly or explicitly. For this reason, the BTV delineation in this work is obtained through the evolution of a fully 3D surface. While some key features of the present system remained unchanged with respect to the previous version [24], such as (i) input is minimal and consist of just one manual delineation on one pet slice (see Section 2.1.), (ii) conversion of PET images into SUV images (see Section 2.1.2), and (iii) an optimal mask is retrieved during the pre-segmentation step, the core approach to segmentation, the initialization strategy and the DA labelling underwent consistent changes. In particular, initialization is now provided in form of a 3D shape and the DA has been re implemented to handle voxels, as opposed to pixels.

Finally, for better comparison, I used the same PET dataset as in Comelli et al. [24] where I demonstrated that the 2D approach already performed better than many commonly employed delineation methods. My results clearly show that both the migration from assembled 2D to full 3D, and the inclusion of tissue classification contributed to improve performance regardless of the anatomical district considered. In particular, in terms of DSC the improvement can be summarized as follows: 90.47 ± 2.36% versus 88.01 ± 4.23% for lung cancer, 88.30 ± 2.89% versus 87.15 ± 3.23% for head and neck cancer, and 90.29 ± 2.52% versus 89.58 ± 2.37% for brain cancer. Moreover, it is worth noting that, with the active surface, no stopping condition is needed and any preferential direction is removed. The converged active surface delivers a final, fully operator-independent 3D segmentation. In addition, I included the tissue classification, which was implemented in 3D as well. In this way, both the evolving shape and the tissue classification included in the energy, and consequently in the shape evolution, exploit cross-slice information that has never been exploited before. Despite (from a surficial search of the literature) the impression that the combination of ML with the active surface approach has been investigated by many authors, most studies have instead kept active surface and ML as independent entities. In contrast, I integrated ML directly in the energy of the active surface approach.

## 5. Conclusions

In this study, I discussed the inclusion of 3D tissue classification into fully 3D segmentation based on the evolution of an active surface. The obtained algorithm outperforms my previous formulation in which the tumor delineation was achieved using a 2D active contour approach. As an immediate advantage, the active surface is capable of segmenting all volume at once. Additionally, I enriched such an algorithm with the inclusion of a machine learning component, which consists of a 3D tissue classification based on discriminant analysis and compared the results with previous work. Noteworthy, both shape evolution and tissue classification can now leverage cross-slice information that was not available before. Based on the reported results, I can claim that migrating the system into 3D yielded a practical benefit justifying the effort to rewrite an existing 2D system for PET imaging segmentation.

## Figures and Tables

**Table 1:** Sensitivities, PPVs, DSCs and HDs for cancer studies using the 3D and 2D algorithms.

	**3D Active Surface with 3D Discriminant Analysis**		
	**Lung**	**Head and Neck**	**Brain**
	Mean ± std	Mean ± std	Mean ± std
**Sensitivity**	90.09 ± 6.50%	86.05 ± 5.70%	89.61 ± 4.29%
**PPV**	91.36 ± 3.89%	91.19 ± 5.30%	91.27 ± 4.52%
**DSC**	90.47 ± 2.36%	88.30 ± 2.89%	90.29 ± 2.52%
**HD**	1.40 ± 0.72	1.33 ± 0.57	1.04 ± 0.49
	**2D Active Contour with 2D Discriminant Analysis**		
	**Lung**	**Head and Neck**	**Brain**
	Mean ± std	Mean ± std	Mean ± std
**Sensitivity**	88.00 ± 5.41%	89.28 ± 5.70%	89.58 ± 3.40%
**PPV**	88.19 ± 4.73%	85.53 ± 5.13%	89.76 ± 3.31%
**DSC**	88.01 ± 4.23%	87.15 ± 3.23%	89.58 ± 2.37%
**HD**	1.53 ± 0.54	1.18 ± 0.39	1.07 ± 0.61

**Table 2:** ANOVA and PCC on the DSC showed statistical differences between 2D and 3D segmentation methods.

Active Surface with Discriminant Analysis	ANOVA *p*-Value	Mean DSC Difference	PCC	PCC *p*-Value
**2D vs. 3D**	0.046	2.45%	0.53	0.000044
